# The Relationship Between Retrospectively Measured Pregnancy Intentions and Women’s Stages of Behavior Change for Contraceptive Use and Effectiveness Level of Contraceptive Method Choice

**DOI:** 10.3390/epidemiologia6040087

**Published:** 2025-12-02

**Authors:** Otobo I. Ujah, Jason L. Salemi, Rachel B. Rapkin, William M. Sappenfield, Ellen M. Daley, Russell S. Kirby

**Affiliations:** 1 Department of Obstetrics and Gynaecology, Federal University of Health Sciences, P.M.B 145, Otukpo 972101, Nigeria; otoboujah@yahoo.com; 2 College of Public Health, University of South Florida, Tampa, FL 33612, USA; jsalemi@usf.edu (J.L.S.); wsappenf@usf.edu (W.M.S.);; 3 Tampa General Hospital, CR 4th Floor, Tampa, FL 33606, USA; 4 Department of Obstetrics and Gynecology, Wellington Regional Hospital, Te Whatu Ora, P.O. Box 793, Wellington 6140, New Zealand

**Keywords:** contraceptive, pregnancy intendedness, method choice, adoption readiness, multilevel analyses

## Abstract

**Background/Objectives**: Unintended pregnancy is linked to an increased risk of subsequent unintended pregnancies, but its impact on contraceptive use and intention remains vastly understudied. This study assessed whether unintended pregnancy independently influences women’s stages of behavior change for contraceptive use and the effectiveness of their chosen contraceptive methods. **Methods**: Using pooled data from three cross-sectional surveys of the Performance Monitoring and Accountability 2020 project in Nigeria, we analyzed responses from 8014 non-pregnant women aged 15–49 years nested within 892 communities. Multilevel multinomial logistic regression accounted for compositional and contextual factors. **Results**: Women with a mistimed pregnancy had higher odds of being in the contemplation stage of behavior change compared to those with an intended pregnancy (adjusted odds ratio [aOR] = 1.59, 95% confidence interval [CI] = 1.13–2.22), with a similar but non-significant trend for unwanted pregnancies (aOR = 1.46, 95% CI = 0.91–2.34). Mistimed and unwanted pregnancies were also linked to higher odds of being in the action stage (aOR = 2.17 and 1.85, respectively). Regarding contraceptive effectiveness, women with a mistimed pregnancy were more likely to use moderately effective methods (aOR = 1.47, 95% CI = 1.02–2.12) and highly effective methods (aOR = 2.45, 95% CI = 1.41–4.26). Unwanted pregnancies showed even stronger associations with highly effective methods (aOR = 4.03, 95% CI = 1.18–13.74). Community-level variability significantly influenced outcomes. **Conclusions**: Together, these findings underscore the importance of person-centered approaches and public health interventions tailored to stages of contraceptive behavior change, targeting both women and communities at high risk of unintended pregnancy.

## 1. Introduction

Unintended pregnancies, resulting from contraceptive failure or nonuse, remain a major public health concern, particularly in sub-Saharan Africa (SSA), where they contribute to adverse maternal and family outcomes [1,2,3,4,5]. Contraceptive use, especially effective methods, offers a cost-efficient means to prevent unintended pregnancies and their consequences [2,6]. Globally, an estimated 120 million unintended pregnancies occurred annually between 2015 and 2019, accounting for 48% of all pregnancies, with SSA recording the highest rates at 91 per 1000 women aged 15–49 years [7]. These estimates highlight the urgent need to expand access to and utilization of effective contraception across the region.

It is well established that personal experiences strongly influence health behavior [8]. Experiencing an unintended pregnancy may influence future reproductive decisions, including the desire to space, delay or limit births, thereby shaping contraceptive motivation and readiness [9,10,11]. Studies suggest that such women may demonstrate greater readiness to adopt modern methods [12], with pathways potentially mediated through emotional, cognitive or contextual mechanisms [11,13].

Multiple factors across individual, household and community levels influence the incidence and recurrence of unintended pregnancies [14]. Further, evidence suggests that women with a history of unintended pregnancy have significantly higher odds of experiencing a repeat unintended pregnancy [15], suggesting a cyclical/bidirectional relationship between contraceptive use and pregnancy intention. Understanding how women’s prior pregnancy experiences shape contraceptive decisions is therefore critical to breaking this cycle.

Despite global evidence, population-based studies exploring this relationship in West Africa remain scarce. Existing studies often overlook behavioral readiness and method effectiveness, limiting their applicability for family planning program design [11,13,16,17,18,19]. Addressing this gap is vital in a region with high fertility, maternal mortality and unmet need for contraception.

**Figure 1:**
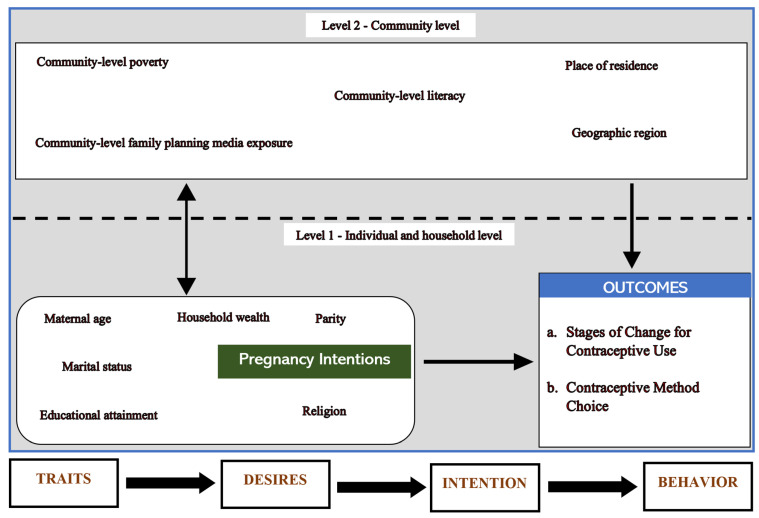
A conceptual framework integrating the TDIB framework, social ecological model and the stages of change (SoC).

To deepen understanding of the multifaceted factors shaping women’s contraceptive decision-making after unintended pregnancy, this study employs a multi-theoretical framework that integrates the Traits–Desires–Intention–Behavior (TDIB) framework, the Social Ecological Model (SEM) and the Stages of Change (SOC) model. The TDIB framework, developed by Miller [20], provides a motivational sequence linking dispositional traits to pregnancy desires, intentions and subsequent contraceptive behaviors. It allows us to conceptualize how psychological and motivational processes translate into fertility regulation decisions [20]. The SEM extends this perspective by situating these individual-level dynamics within a broader ecological context, recognizing that contraceptive behaviors are shaped by interpersonal, community and societal factors [21,22]. Complementing both, the SOC model captures the temporal and motivational progression from precontemplation to action and maintenance, offering a nuanced lens to assess readiness for contraceptive use [23,24,25]. Together, these frameworks provide a holistic approach to understanding how individual motivations and contextual factors interact to influence post-pregnancy contraceptive behaviors (Figure 1).

This study examines the association of retrospective pregnancy intention with readiness to adopt contraception and the likelihood of choosing more effective methods among nonpregnant women of reproductive age. Specifically, we ask: (1) To what extent is a recent unintended pregnancy associated with stages of behavioral change in contraceptive use? and (2) How does such experience influence the effectiveness level of contraceptive method choice? Grounded in behavioral theory, this study advances understanding of the link between pregnancy intention and subsequent contraceptive behavior, informing strategies to enhance family planning services and contraceptive uptake in SSA.

## 2. Materials and Methods

### 2.1. Study Design and Data Source

This study used data from the Performance Monitoring and Accountability (PMA) surveys conducted in Nigeria. The PMA surveys are implemented either annually or semi-annually to monitor key indicators in family planning and reproductive health. Using smartphone-assisted technology, the survey gathers information from households, women aged 15–49 years and service delivery points. Data are collected by trained female resident enumerators through a multi-stage stratified cluster sampling design that ensures representativeness at both national and subnational levels.

In brief, the Nigeria PMA survey follows a three-stage cluster sampling approach. States are first selected from each geopolitical zone using probability proportional to size (PPS), followed by the selection of enumeration areas within states from the National Population Commission’s master sampling frame, and finally, random selection of 35–40 households per cluster. Eligible women in each household were interviewed by trained female enumerators. Response rates remained consistently high across survey rounds, exceeding 97% among both households and eligible women. Ethical approval for the survey was obtained from the Johns Hopkins Bloomberg School of Public Health and the National Health Research Ethics Committee of Nigeria, with informed verbal consent obtained from all participants. Further details of the survey design, sampling procedures and data collection protocols are publicly available at https://www.pmadata.org (assessed 15 January 2025) and in prior methodological publications [26]. The protocol for this study was reviewed and deemed exempt by the Institutional Review Board of the University of South Florida (IRB ID: STUDY005438).

### 2.2. Sample

**Figure 2:**
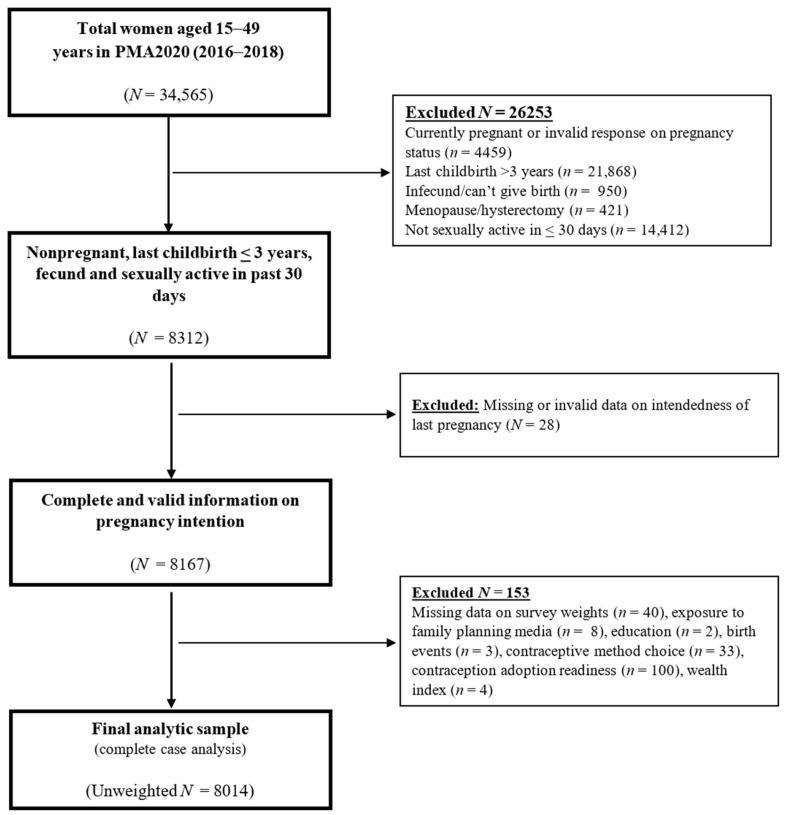
Graphical Presentation of Study Sample Selection, Nigeria, Performance Monitoring for Accountability Survey (PMA2020) Rounds 3, 4 and 5, 2016–2018.

In this study, we pooled data from the 2016, 2017 and 2018 rounds of the PMA2020 survey. The goal was to increase the sample size and improve the statistical power of our study. We restricted our analyses to nonpregnant women aged 15–49 years with a last childbirth within the 3 years prior to the survey, who provided data on pregnancy intention, stages of behavior change for contraceptive use and contraceptive method choice and were sexually active in the 30 days prior to the survey. Women who reported menopause/hysterectomy, infertility, were missing survey weight data, or did not provide valid responses to any of the covariates were excluded from the analyses (Figure 2). Based on the eligibility criteria for this study, an unweighted sample of 8014 women were included in the analyses.

### 2.3. Measures

#### 2.3.1. Independent Variable

The main independent variable was self-reported pregnancy intention based on retrospective recollection of their desire to become pregnant prior to conception. During the survey, women of childbearing age (15–49 years) were asked “At the time you became pregnant, did you want to become pregnant then, did you want to wait until later, or did you not want to have any children at all?”. The response options were “then”, “later” and “not at all”. For this study, pregnancy intention (for their most recent pregnancy within the previous 3 years) was operationalized as a 3-level exposure variable. Similar to the approach used in a prior study [27], women who responded that they wanted to be pregnant then were classified as “intended”. Women who said they wanted to be pregnant later were classified as “mistimed” and those who did not want to be pregnant at all were classified as “unwanted”.

#### 2.3.2. Dependent Variable

We analyzed two main outcome measures related to contraceptive behaviors (1) stage of behavior change for contraceptive use and (2) contraceptive method effectiveness level. The stages of change for contraceptive use were operationalized as a 3-level variable based on the transtheoretical model and was derived from two specific questions asked during the survey. First, eligible female respondents were asked, “Are you or your partner currently doing something or using any method to delay or avoid getting pregnant”. For the purposes of this study, those who answered “yes” were identified as being at the action stage of change, indicating that they were already taking action or maintaining contraceptive use. On the other hand, those who answered “no” were further asked, “You said that you are not currently using a contraceptive method. Do you think you will use a contraceptive method to delay or avoid getting pregnant at any time in the future?” Among this subgroup, those who answered “no” were identified as being at the precontemplation stage, indicating they were not considering contraceptive use (i.e., not using contraception and is not intending to change this behavior). Conversely, those who answered “yes” were at the contemplation stage, suggesting they were contemplating the use of contraceptive methods in the future (i.e., similar precontemplation, however, they had intentions of using contraception in the future).

**Figure 3:**
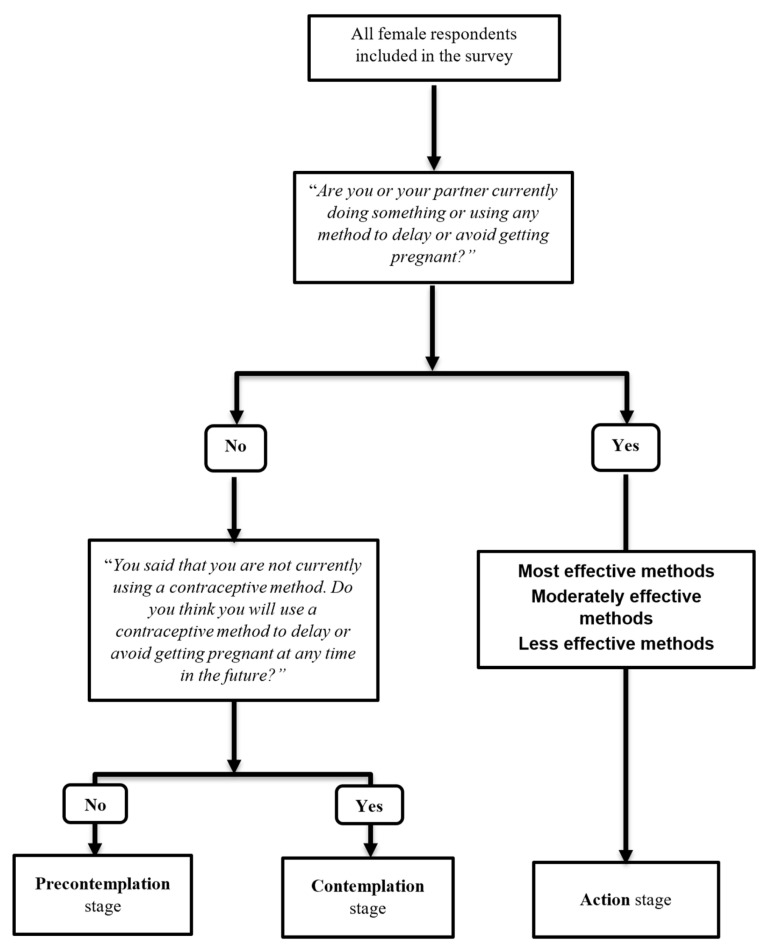
Operationalization of stages of behavior change for contraceptive use and contraceptive method effectiveness level.

We defined contraceptive method effectiveness level as a 4-level outcome variable. This was ascertained from two survey questions. The first question asked, “Are you or your partner currently doing something or using any method to delay or avoid getting pregnant?”. Women who answered “no” were classified as using no method used. Women who answered “yes” were asked “Which method or methods are you using?” to ascertain the specific method of contraception being used and classify participants as into users of most effective (such as intrauterine devices, implants, or sterilization), moderately effective (such as injectables, pills, or diaphragms) and less effective methods (such as condoms, other barrier/traditional methods, emergency contraception, standard days method, or lactational amenorrhea method) [28] (Figure 3). Women who were using more than one contraceptive method were classified according to the most effective method based on its typical use failure rate (<1%), consistent with prior studies [28,29].

#### 2.3.3. Control Variables

The selection of covariates for this study was determined through a comprehensive literature review, their biological plausibility in the exposure–outcome relationship, and the availability of variables in the survey data that were consistently measured across all survey rounds. Following the social ecological model [21], these variables were classified into two levels: individual/household and community factors. At the individual and household levels, the following factors were considered: maternal age at the time of the survey (15–24 years, 25–34 years, and 35 years or older), level of educational attainment (less than secondary, secondary, higher than secondary), parity (low (1–2), average (3–4) and high (5+)), marital or cohabiting status (single, married/cohabiting), and exposure to FP mass media (exposed or not exposed), determined based on women reporting exposure to FP messages through at least one media channel, such as radio, television, newspapers, billboards/posters, magazines, brochures/leaflets, or voice/text messages and household wealth index (poorest, poorer, middle, richer, richest). We also considered survey characteristics such as the PMA2020 survey round (Round 3/2016, Round 4/2017 and Round 5/2018).

Primary sampling units or clusters are used as proxies for communities in the Performance Monitoring for Action survey data. However, community-level factors were not directly measured during the survey, except for the place of residence (urban or rural). Therefore, community-level characteristics were derived by aggregating individual-level factors within their respective clusters. The derived variables include the community-level literacy (percentage of women with at least a secondary level of education within a community), community-level poverty (percentage of women residing below the middle household income quintile within a community), and community-level exposure to FP media (percentage of women exposed to at least one form of FP mass media within a community).

### 2.4. Statistical Analysis

#### 2.4.1. Descriptive Analysis

Data analyses were performed using SAS version 9.4 while figures/plots were prepared using R version 4.2.0. We performed a complete case analysis by including only participants for whom all variables of interest were collected. Weighted descriptive statistics were generated using relevant SAS procedures to calculate valid estimates of standard errors (SE) based on the survey design. This involved computing means for continuous variables using PROC SURVEYMEANS and percentages for categorical variables using PROC SURVEYFREQ. These descriptive statistics are reported separately for the two dependent variables to provide background characteristics of survey respondents. The differences between various outcome groups were examined using the Chi-square test.

#### 2.4.2. Multilevel Model Building Strategy

To account for the polytomous nature of the dependent variables, which were both measured on nominal scales, and considering the hierarchical structure of the data with *i* women (level 1) nested within *j* communities (level 2), series of weighted multilevel multinomial logistic regression models were fitted. The purpose of these models was to examine the effect of pregnancy intention on each outcome, adjusting for contextual and compositional factors. We also ran separate analyses for each dependent variable. The SAS PROC GLIMMIX procedure with pseudo-maximum-likelihood approach and multinomial probability distribution with the GLOGIT link function were used for both study outcomes. Furthermore, individual-level (Level 1) weights were scaled to reduce the bias in the variance parameter estimator [30,31].

The estimates of the fixed effects are computed as unadjusted and adjusted odds ratios and 95% confidence intervals for associations between pregnancy intention and women’s readiness to use contraception and contraceptive method effectiveness level. We tested the hypotheses for this study by fitting four 2-level random intercept only models in addition to the null model. The null model did not include any level 1 or level 2 predictors, only random intercepts. Model I included only the pregnancy intention variable; the second model (Model II) included the Model I with individual-level predictors. Model III included Model I with community-level variables. In the full model (Model IV), individual-level and community level predictors were included. Table 1 shows the model building strategy employed in this study. For all models fitted, level 2 continuous predictor variables (community poverty, community literacy and community FP media exposure) were grand-mean centered.

##### Fixed Effects (Measures of Association)

The general equation of the random intercept two-level multinomial logistic regression model used for analysis of predictors of the nominal outcomes with J categories, takes the form:

Log [π*_ij_*/π*_iJ_*] = *β*_0_^(*s*)^
*+ β*_1_^(*s*)^X_1*ij*_ + *β*_2_^(*s*)^X_2*ij*_ + … + *β_(_*_1 − *k*)_^(*s*)^*X*_(1 − *k*)*ij*_
*+ u_j_*^(*s*)^*,*

where *s* represents the specific polytomous outcome (in this case, stages of behavior change for contraceptive use and contraceptive method effectiveness level).

*β*_0_^(*s*)^ are the fixed regression intercepts associated with the specific outcome, *s*.

*X*_(1 − *k*)*ij*_ are K explanatory variables defined at the individual and community level, where K = 1 − k.

*β*_(1 − *k*)_^(*s*)^ are the associated usual regression parameter estimates associated with the specific outcome, *s*.

*uj*^(*s*)^ are the community-level residuals specific to each outcome *s*, assumed to be normally distributed with mean 0 and variance *σ_u_*^2(*s*)^. These provide estimates for the variation in the stages of behavior change for contraceptive use and contraceptive method effectiveness level.

#### 2.4.3. Random Effects (Measures of Variation)

Both Intraclass Correlation Coefficient (ICC) and Proportional Change in Variance (PCV) were computed to evaluate between-cluster variations. For logistic regression models, the variance at the within-community level can be represented by the variance of the standard logistic distribution. By utilizing the logistic distribution variance of approximately 3.29, we computed the intraclass correlation coefficients (ICC) using the following equation:

ICC= τ_00_/(τ_00_ + 3.29),

where τ_00_ is the between-community variance.

Different models were compared using measures of goodness of fit, including the −2 log likelihood, the Akaike information criterion (AIC), and the Bayesian information criterion (BIC). To assess the proportion of community variability accounted for by the variables in models I-IV for each outcome, we computed the PCV. This was calculated by comparing the τ_00(0)_ for models I-III to that of the unconditional model [τ_00(0)_ − τ_00(n)_/τ_00(0)_].

## 3. Results

### 3.1. Characteristics of the Sample

The mean age of respondents was 28.5 years (SD = 6.8), with nearly half aged 25–34 years. As shown in Table 2, most women had secondary level of education, were married or cohabiting and of low parity. Over half resided in poor households, and about two-thirds reported exposure to at least one form of FP mass media. At the community level, nearly half lived in urban areas, with wide variation across communities in household poverty, women’s education, and FP media exposure an average of 53.2% of women lived in poor households (range: 0–100%). The proportion of women with at least secondary education ranged from 0% to 44.1%, while exposure to FP mass media averaged 14.6% (range: 0–42.8%).

As shown in Table 2, approximately 5666 women (70.8%) reported that their pregnancy was intended, while 23.1% reported that their pregnancy was mistimed, and (6.1% reported that their pregnancy was unwanted. There were no significant differences in exposure to FP mass media across the different pregnancy intention categories. However, for all the other sociodemographic and reproductive health characteristics, there were significant differences with pregnancy intention status (*p* < 0.05).

**Figure 4:**
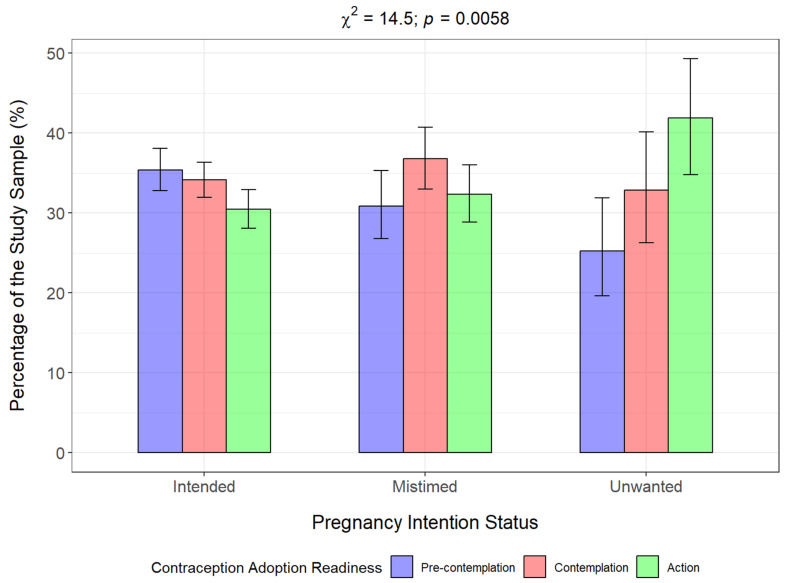
Stages of change in contraceptive use behavior among women, by pregnancy intention.

Regarding stages of behavior change for contraceptive use, 3133 (33.7%) were at the precontemplation stage, 2696 (34.7%) were at the contemplation stage while 2185 (31.6%) were at the action stage. Figure 4 shows a plot of the women based on their stage of contraceptive adoption readiness, categorized by their pregnancy intention status. The proportion of women who were at the precontemplation stage was highest among women who reported their pregnancy as intended (35.4%), while the proportion of women who were at the contemplation stage was highest among those who reported their recent pregnancy as mistimed (40.8%). The highest proportion of women in the action stage was among those who reported their recent pregnancy as unwanted (49.3%). The bivariable analysis revealed statistically significant associations between women’s pregnancy intention and their readiness to adopt a contraceptive method (Rao-Scott χ^2^ = 14.5; *p* = 0.0058).

**Figure 5:**
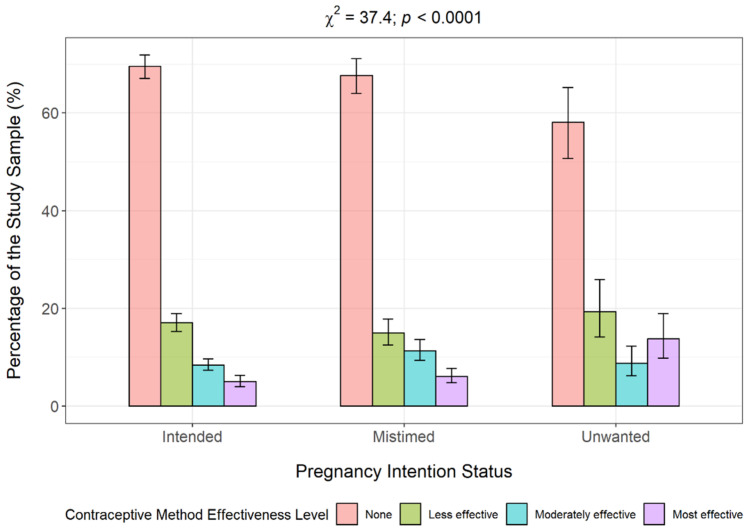
Contraceptive method effectiveness level by pregnancy intention.

Overall, more than two-thirds (68.4%, *n* = 5829) women were not using a contraceptive method. Approximately 16.7% were using the less effective methods, 9.1% were using moderately effective methods, and 5.8% were using a most effective contraceptive method. Figure 5 shows a plot of the effectiveness level of women’s contraceptive method choice by pregnancy intention status. The proportion of women using a less effective and a most effective method was highest among women who reported their recent pregnancy as unwanted pregnancy (19.4% and 13.8%, respectively), relative to those with an intended and mistimed pregnancy. The proportion of women using a moderately effective method was highest among women who reported their recent pregnancy as mistimed (11.3%). Notably, approximately two-thirds (65.7%) of women reporting previous mistimed or unwanted pregnancy (*n* = 2348) were not using a method of contraception. The bivariable analysis showed a significant association between pregnancy intention and the effectiveness level of their contraceptive metho choice (Rao-Scott χ^2^ = 37.4; *p* < 0.0001).

**Figure 6:**
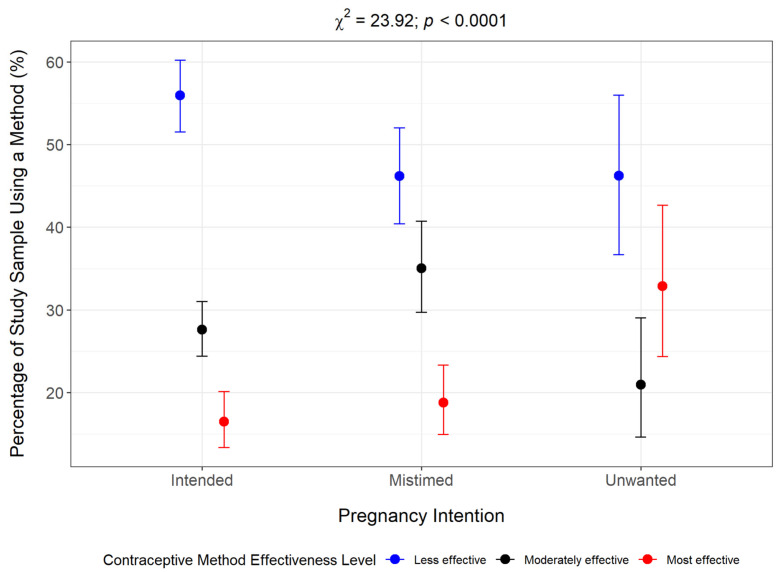
Contraceptive method effectiveness level grouped by pregnancy intention status among women in action stage.

A further analysis of the bivariable relationship between pregnancy intention and contraceptive method effectiveness level but excluding women not using a method revealed that most women were using a less effective method (52.8%), 28.8% were using a moderately effective method, while 18.3% were using a most effective method (Figure 6). Among the different pregnancy intention categories, the proportion of women using a less effective method was highest for women who reported an intended pregnancy (55.9%) compared to women with unintended pregnancies (mistimed: 46.2%; unwanted: 46.20%). Conversely, a higher percentage of women with unintended pregnancies were using a most effective method (mistimed: 23.4%; unwanted: 42.6%) compared to women who had an intended pregnancy. The bivariable analysis suggests a significant association between pregnancy intention and contraceptive method effectiveness level (Rao-Scott χ^2^ = 23.92; *p* < 0.0001).

### 3.2. Results of Multilevel Regression Analyses

#### 3.2.1. Stages of Behavior Change for Contraceptive Use

##### Unconditional Model

Table 3 shows the result of the multilevel analysis for the associations between pregnancy intention and women’s readiness to use contraception. For the fixed effects, the null model indicates that the log odds of being in the contemplation stage at a typical community, where the random effect on the logit scale is zero, were 0.11, resulting in a predicted probability of 0.34. Similarly, the log odds of being in the action stage at a typical community were 0.15, resulting in a predicted probability of 0.35. For the reference category (precontemplation stage), the predicted probability is 0.31. The results also show a statistically significant amount of variability in the log odds of being in the contemplation stage (τ00 = 1.82, SE = 0.32, z(1816) = 5.55, *p* < 0.0001) and in the action stage (τ00 = 3.34, SE = 0.40, z(1816) = 8.36, *p* < 0.0001) between the communities.

For being in the contemplation versus precontemplation stage, the ICC is 0.35. This indicates approximately 35.6% of the variability in being in the contemplation stage was accounted for by the communities in the study sample, while the remaining 64.4% of the variability could be due to systematic differences between women or other unidentified factors. The ICC for being in the action versus precontemplation stage was estimated to be 0.50 suggesting that about 50.4% of the variability in being in the action stage can be explained by the communities, leaving 49.6% of the variability to be accounted for by systematic differences between women or other unidentified factors.

##### Conditional Models

The results also showed that pregnancy intention was positively associated with women’s readiness to use a contraceptive method (Table 3). The model fit indices showed that Model IV was superior to the other models (AIC = 18,329.02, BIC = 18,587.87). Based on this model which adjusted for individual-/household-level and community-level covariates, women who had a recent mistimed pregnancy had 59% higher odds of being in the contemplation stage (aOR = 1.59, 95% CI = 1.13–2.22) and at least a twofold higher odd of being in the action stage (aOR = 2.17, 95% CI = 1.52–3.11), compared to women whose pregnancy was intended. Although women who reported having an unwanted pregnancy had an 82% significantly higher odds of being in the action stage compared to women who had an intended pregnancy (aOR = 1.85, 95% CI = 1.18–2.91), this difference was not statistically significantly for women with unwanted vs. intended pregnancy and being in the contemplation stage (aOR = 1.46, 95% CI = 0.74–2.89).

Table 3 also presents the adjusted random effects, which include the explained variance (represented by the PCV), for Models I–IV. In comparison to the null model, the inclusion of pregnancy intention alone accounted for a reduction of 2.98% and 4.63% in the variance of being in the contemplation and action stages of contraceptive adoption readiness, respectively. In the best fitting model (Model IV), relative to the null model, the inclusion of individual-/household- and community-level predictors accounted for 29.76% and 59.16% of the variability in being in the contemplation and action stages of behavior change related to contraceptive use, respectively.

#### 3.2.2. Contraceptive Method Effectiveness Level

##### Unconditional Model

Table 4 shows the result of the analysis fitting a logistic regression model with a random intercept examining the association between pregnancy intention and contraceptive method effectiveness level. The fixed effect estimates of the null model show that the log odds of using less effective methods were −2.23, resulting in a predicted probability of 0.09. The log odds of using a moderately effective method were −2.31, resulting in a predicted probability of 0.08 while the log odds of using a most effective method in a typical community were −3.24, resulting in a predicted probability of 0.03. The probability of not using a contraceptive method was 0.80.

The null, random effects model showed a statistically significant amount of variability in the log odds of using a less effective method (τ_00_ = 4.92, SE = 0.65, z(2724) = 7.52, *p* < 0.0001), a moderately effective method (τ00 = 2.02, SE = 0.44, z(2724) = 4.56, *p* < 0.0001) and a most effective method (τ_00_ = 3.05, SE = 0.56, z(2724) = 5.45, *p* < 0.0001) between the communities.

The ICC for using a less effective method was 0.60, indicating that approximately 60% of the variability in using a less effective method was accounted for by the communities in the study sample, while the remaining 40% of the variability could be attributed to individual differences or other unidentified factors. The estimated ICC for a moderately effective method versus no method was 0.38, indicating that approximately 38% of the variability in using a moderately effective method was accounted for by the communities, while the remaining 62% of the variability could be accounted for by individual differences or other unidentified factors. The estimated ICC for a most effective method versus no method was 0.48, indicating that approximately 48% of the variability in using a most effective method was accounted for by the communities in our study, while the remaining 52% of the variability could be attributed to individual differences or other unidentified factors.

##### Conditional Models

The results of the fixed effects for the association between pregnancy intention and contraceptive method effectiveness level are presented in Table 4. The results of the model fit indices suggest that Model IV was the best fitting model for our data and therefore was used to determine the effect of pregnancy intention. In the fully adjusted model (Table 4, Model IV), women who reported a mistimed pregnancy had a 47% significantly higher odds of using a moderately effective method (aOR = 1.47, 95% CI = 1.02–2.12), and a more than twofold higher odds of using a most effective method (aOR = 2.45, 95% CI = 1.41–4.26), compared to women who had an intended pregnancy. There was no significant difference in the association between having a mistimed vs. intended pregnancy and the use of a less effective method. Women who had an unwanted pregnancy, compared to their counterparts who had an intended pregnancy were 6.19 times more likely to use a most effective contraceptive method (95% CI = 2.04–18.83).

Table 4 also presents the adjusted random effects, which include the explained variance for Models I-IV. In comparison to the null model, the inclusion of pregnancy intention alone accounted for a reduction of 1.31%, 4.05% and 15.16% in the variability in using a less, moderately and most effective contraceptive method, respectively. In the best fitting model (Model IV), compared to null model, the incorporation of level-1 and level-2 characteristics in the full Model accounted for a 63.68%, 34.14% and 42.20 of the variability for the use of a less, moderately and most effective contraceptive method.

## 4. Discussion

Using a theory-informed and multilevel analytical approach, this study examined whether and to what extent the intendedness of a recent pregnancy influences contraceptive use and readiness to adopt contraception among women in Nigeria. Among a nationally representative sample of nonpregnant women of reproductive age, most women who had experienced a mistimed or unwanted pregnancy were not currently using contraception. Our findings revealed significant associations between pregnancy intentions, stages of behavioral change for contraceptive use, and the effectiveness level of chosen contraceptive methods, even after controlling for compositional and contextual factors. Importantly, women’s readiness for contraceptive adoption and their likelihood of using more effective methods varied by the degree of pregnancy intendedness. These results suggest that women who recently experienced an unintended pregnancy are generally more motivated to prevent recurrence, as reflected by their stage of readiness and their choice of contraceptive method. Additionally, substantial variability across communities—captured by the random intercepts—was observed for both contraceptive readiness and method effectiveness, though these community-level differences diminished after adjusting for individual, household and contextual variables.

Collectively, these findings are consistent with the social ecological model, emphasizing the influence of contextual factors on women’s contraceptive readiness and decision-making. The observed positive association between unintended pregnancy and subsequent contraceptive use is consistent with previous research. Zimmerman and colleagues [32], for example, reported higher odds of contraceptive adoption among women who experienced mistimed pregnancies, particularly in the first postpartum year. Similar evidence from sub-Saharan Africa (SSA) shows that women with unintended pregnancies are more likely to use contraception subsequently [12,17]. Studies using the U.S. National Survey of Family Growth (NSFG) have likewise found that women reporting mistimed or unwanted births were nearly twice as likely to adopt a highly effective method as those whose births were intended [9]. Together, this body of evidence—including the present study—highlights that contraceptive intentions and behaviors differ markedly depending on whether the most recent pregnancy was mistimed or unwanted.

Our findings further suggest that the implications of mistimed and unwanted pregnancies for contraceptive behavior are distinct. A mistimed pregnancy may strengthen motivation for spacing and the use of reversible long-acting methods such as IUDs or implants, whereas an unwanted pregnancy may indicate completed fertility, making permanent methods like tubal ligation more appropriate. Recognizing these differences is crucial for tailoring FP counseling to women’s reproductive goals and stage of readiness. These programmatic distinctions have profound public health implications, underscoring the importance of personalized and context-specific reproductive health services.

By jointly examining motivational and behavioral dimensions of contraceptive change, this study extends understanding of how unintended pregnancy shapes subsequent contraceptive intentions and behaviors. The findings reinforce the value of identifying women’s stages of readiness for contraceptive behavior change and tailoring interventions accordingly. Women with a recent unintended pregnancy represent a priority group, differing in motivation, readiness and contextual barriers and thus require targeted interventions that not only expand access but also address community-level determinants influencing contraceptive decision-making.

Our operationalization of pregnancy intention relied on a timing-based measure, which captures primarily the cognitive dimension of intendedness. However, prior literature recognizes cognitive, affective and contextual components of pregnancy intention [33,34]. Future research could therefore explore how these dimensions jointly influence stages of behavioral change for contraceptive use among women with unintended pregnancies, identifying which are most salient for stage-matched interventions to prevent recurrence.

Beyond categorizing women into motivational and volitional phases, we also quantified readiness through contraceptive method effectiveness, distinguishing intention from actual behavior. This distinction is critical as intention alone may not translate into action. Consistent with the Theory of Planned Behavior [35], intention remains a key determinant of behavior, but stage-tailored interventions—addressing where women are along the precontemplation-to-maintenance continuum—are likely to yield stronger, more sustained outcomes. Interventions that move women from precontemplation to contemplation or from contemplation to action can improve both short- and long-term contraceptive adoption.

Despite the strengths of a nationally representative dataset and a theory-driven approach, our study has several limitations. First, its cross-sectional design limits causal inference; while consistent with a directional hypothesis, temporal ordering cannot be established. Second, the survey was not specifically designed to test the Stages of Change model, constraining assessment of temporal progression between stages. Consequently, while we classified readiness at a single time point, we could not observe transitions—progression, regression or stability—over time. While the SOC model provided a useful framework for understanding readiness for contraceptive adoption, it is important to acknowledge ongoing scholarly debates regarding its conceptual limitations. Critics have argued that the TTM from which the SOC model derives, tends to oversimplify behavioral change into discrete and sequential stages, whereas in reality, change may be more fluid, overlapping and context-dependent, as Robert West (2005) contends [36]. Third, we were unable to include other key components of the transtheoretical model such as processes of change and decisional balance, which could have strengthened interpretive validity. Additionally, our adaptation of the SOC model into three broad stages—based on available survey items—may have introduced some conceptual overlap, potentially resulting in stage misclassification. Furthermore, we did not account for contraceptive use prior to the recent pregnancy, which could have provided valuable insights into whether unintended pregnancies resulted from contraceptive failure. Our focus on nonpregnant women also limits generalizability, as excluding women whose unintended pregnancies ended in abortion or miscarriage may introduce selection bias. Research shows that the outcome of a pregnancy may influence mothers’ reports of pregnancy intention, with a tendency to switch from reporting pregnancies as unintended to intended after birth, thereby underestimating unintended pregnancies in retrospective surveys [37]. These women may differ substantially in contraceptive motivation and behavior. Similarly, excluding sexually inactive women (those not active in the 30 days preceding the survey) might have omitted individuals practicing abstinence as a deliberate pregnancy prevention strategy, potentially skewing results toward those with immediate contraceptive needs and inflating associations between behavioral stage and contraceptive consideration. Finally, as pregnancy intention and contraceptive use were self-reported, recall bias remains possible.

Our findings carry important implications for reproductive health programming. Interventions should classify women with recent unintended pregnancies according to their stage of change, tailoring counseling and support to their level of readiness and contraceptive intention before implementing stage-matched interventions. Recognizing differences between mistimed and unwanted pregnancies is essential: women desiring spacing require accessible, reversible methods, while those wishing to limit births may need counseling on long-acting or permanent options. Integrating these distinctions into prenatal, postnatal and FP services can promote person-centered contraceptive counseling, responsive to women’s lived experiences and reproductive goals.

Future research should incorporate partner intentions and examine couple-level concordance or discordance in pregnancy intentions and its effects on contraceptive behavior. Longitudinal designs would further clarify causal pathways and progression through stages of change. Additionally, exploring how pregnancy outcomes, such as abortion, miscarriage or live birth, moderate the relationship between pregnancy intention and contraceptive behavior will refine understanding and inform targeted interventions.

Finally, to achieve sustained improvements in contraceptive uptake and reduce repeat unintended pregnancies, health systems must ensure service readiness, expanding the availability, accessibility, and affordability of a broad range of effective contraceptive methods. Tailoring counseling to readiness stage and pregnancy intention, rather than applying a uniform approach, can substantially enhance the effectiveness and person-centeredness of family planning programs in Nigeria and similar contexts.

Taken together, our findings underscore that reproductive autonomy is dynamic, shaped by prior experiences, contextual conditions and motivational readiness, thereby highlighting the value of behavioral-stage frameworks for person-centered family planning.

## 5. Conclusions

A considerable majority of women who experienced a recent unintended pregnancy were not using contraception. Further, our results suggest that although women differed in their readiness for contraceptive adoption and their choice of contraceptive method depending on their level of pregnancy intendedness, experiencing a recent unintended pregnancy may be a strong motivating factor to avoid future unintended pregnancies, thus necessitating the decision to use effective and reliable contraceptive methods. Therefore, public health efforts to prevent unintended pregnancy should consider women’s specific needs and readiness for contraceptive adoption. Additionally, classifying women with recent unintended births according to their stages of readiness for contraceptive use will ensure effective implementation of multilevel multicomponent and stage-matched interventions to reduce the risk of repeat unintended pregnancy.

## Figures and Tables

**Table 1:** Exposure variables used in multilevel multinomial logistic regression models.

Null Model^†^	Model 1	Model 2	Model 3	Model 4
Intercept-only model	Pregnancy intention	Level 1 model	Level 2 model	Full multilevel model
No predictors, just random effects for the intercept	Pregnancy intention	Pregnancy intention	Pregnancy intention	Pregnancy intention with all individual- and community-level predictors (i.e., Model 2 + Model 3)
		PMA2020 survey round/year	Place of residence	
		Maternal age	Community-level literacy	
		Education	Community-level poverty	
		Marital/cohabiting status	Community-level family planning media exposure	
		Fertility intentions	Community-level modern contraceptive use	
		Pregnancy intention		
		Education		
		Birth events		

^†^ Output used to calculate ICC—provides information on how much variation in the outcome exists between level-2 units.

**Table 2:** Characteristics of the study sample, overall and according to pregnancy intention status, Performance Monitoring for Action 2020 (PMA2020) survey, Nigeria, 2016–2018.

Characteristics	Study Population		Intended Pregnancy			Unintended Pregnancy						*p*
						Mistimed			Unwanted			
	*N* ^†^	%^‡^	*n* ^†^	%^‡^	95% CI	*n* ^†^	*%* ^‡^	95% CI	*n* ^†^	%^‡^	95% CI	
Sample size	8014	100	5666	70.88	68.71–72.96	1913	23.07	21.09–25.18	435	6.05	5.19–7.03	
**Individual-/household-level variables**												
PMA survey round/year												
Round 3/2016	2614	32.44	1746	30.51	26.45–34.89	705	37.72	31.67–44.42	163	34.24	27.07–43.69	0.02
Round 4/2017	2719	33.75	1960	33.76	29.46–38.34	619	33.63	27.93–39.85	140	34.24	26.61–42.79	
Round 5/2018	2681	33.80	1960	35.74	31.15–40.59	589	28.65	23.49–34.42	132	30.83	23.21–39.66	
Age, mean (SE), years	28.50 (6.78)		28.19 (6.66)			28.66 (6.62)			32.83 (7.97)			
15–24	2342	24.97	1722	25.63	28.81–27.54	521	23.49	20.96–26.22	99	22.85	17.67–29.01	<0.0001
25–34	3949	52.0.4	2834	53.63	51.69–55.54	968	52.30	49.25–55.34	147	32.39	26.94–38.39	
35–49	1723	22.99	1110	20.74	19.18–22.41	424	24.21	21.41–27.26	189	44.75	37.54–52.19	
Education												
Less than secondary	4373	46.38	3047	44.48	41.49–47.51	1098	52.76	48.14–57.33	228	44.45	37.12–51.82	<0.001
Secondary	2681	37.12	1868	37.03	34.66–39.47	638	35.59	31.74–39.65	175	43.93	36.74–51.39	
Higher than secondary	960	16.49	751	18.49	16.43–20.73	177	11.65	9.46–14.26	32	11.72	7.71–17.42	
Marital/cohabiting status												
Single	168	2.59	88	1.85	1.41–2.43	48	3.25	2.32–4.56	32	8.83	5.47–13.93	<0.0001
Married/cohabiting	7846	97.40	5578	98.15	97.57–98.59	1865	96.75	95.45–97.69	403	91.17	86.07–94.53	
Parity												
Low (1–2)	3080	40.45	2380	44.97	43.03–46.93	588	29.71	26.76–32.83	112	28.57	22.27–35.84	<0.0001
Average (3–4)	2513	32.81	1805	33.48	31.85–35.15	639	34.79	31.99–37.69	69	17.36	12.92–22.92	
High (5+)	2421	26.74	1481	21.55	19.95–23.25	686	35.50	32.27–38.88	254	54.07	46.09–61.85	
Exposure family planning media												
Not exposed	2534	32.66	1825	32.25	29.67–35.17	565	33.89	29.75–38.29	144	31.65	25.81–38.13	0.71
Exposed	5480	67.33	3841	67.64	64.83–70.34	1348	66.11	61.71–70.25	291	68.36	61.87–74.19	
Household wealth index												
Poorest	3131	30.65	2210	29.14	25.93–32.58	782	36.95	31.84–42.38	139	24.26	17.85–32.08	0.0001
Poorer	1839	21.48	1251	20.83	18.57–23.29	467	22.93	19.67–25.81	121	24.83	19.04–31.69	
Middle	1147	15.46	798	15.50	13.75–17.44	276	14.93	12.32–17.97	73	16.97	12.36–22.84	
Richer	1021	16.35	715	16.70	15.05–18.49	244	14.61	12.08–17.55	62	18.82	13.89–24.98	
Richest	876	16.35	692	17.82	15.59–20.28	144	10.92	8.64–13.72	40	15.12	9.82–22.56	
**Community-level variables**												
Place of residence												
Rural	4829	52.07	3392	50.41	46.26–54.56	1184	56.87	50.98–62.57	253	53.28	44.83–61.55	0.04
Urban	3185	47.93	2274	49.59	45.44–53.74	729	43.13	37.44–49.02	182	46.72	38.46–55.17	
Geographic region												
North Central	1021	12.95	742	13.25	11.29–15.49	228	11.75	9.24–14.83	51	14.11	8.77–21.92	<0.0001
North East	623	14.12	383	12.49	10.56–14.72	205	19.54	15.34–24.56	35	12.48	7.32–20.48	
North West	4341	33.72	3050	33.11	30.03–36.33	1088	37.59	32.76–42.68	203	26.21	20.34–33.06	
South East	564	8.35	413	8.77	7.39–10.37	109	6.28	4.84–8.11	42	11.29	7.73–16.19	
South South	621	13.03	394	12.02	9.94–14.48	162	13.47	10.67–16.88	65	23.11	16.61–31.20	
South West	844	17.83	684	20.36	18.11–22.82	121	11.36	8.74–14.64	39	12.81	9.20–17.5	
Community literacy, mean (SE) ^a^, %	12.9 (0.4)		13.3 (0.4)			11.5 (0.5)			15.3 (0.6)			
Community poverty, mean (SE) ^b^, %	53.2 (1.5)		51.4 (1.6)			60.2 (2.2)			47.4 (2.8)			
Community FP media exposure, mean (SE) ^c^, %	14.6 (0.3)		14.7 (0.3)			13.8 (0.4)			15.7 (0.5)			

^†^ Unweighted sample size. ^‡^ Weighted column percentage. Percentage may not sum to 100 due to missing values or rounding. Abbreviations: SE, standard error; CI, confidence interval. *p* values are based on Rao-Scott Chi-Square test. ^a^ Percentage of women in PSU living in households in the poorest or poorer wealth quintiles. ^b^ Percentage of women in PSU who have less than a secondary level of educational attainment. ^c^ Percentage of women in PSU who are exposed to at least one form of family planning mass media.

**Table 3:** Odds ratios from multilevel multinomial logistic regression analyses assessing stages of change in contraceptive use behavior, by individual/household- and community-level factors, according to the model.

	Null Model ^a^		Model IV ^e^	
	Contemplation	Action	Contemplation	Action
**Fixed effects intercept** ^‡^	1.12	1.16	1.09	1.16
			**aOR [95% CI]**	
PMA survey round/year				
Round 3/2016			Reference	Reference
Round 4/2017			1.32 (0.85–2.04)	1.16 (0.77–1.76)
Round 5/2018			1.08 (0.72–1.62)	1.17 (0.78–1.77)
**Individual-/household-level variables**				
Pregnancy intention				
Intended			Reference	Reference
Mistimed			1.59 (1.13–2.22) **	2.17 (1.52–3.11) ***
Unwanted			1.46 (0.74–2.86)	1.85 (1.18–2.91) **
Maternal age, y				
15–24			Reference	Reference
25–34			2.15 (1.11–4.18) *	2.47 (1.39–4.38) **
35–49			1.19 (0.67–2.13)	1.79 (0.99–3.22)
Education				
Less than secondary			Reference	Reference
Secondary			2.04 (1.38–3.03) **	1.57 (1.09–2.24) *
Higher than secondary			2.24 (1.15–4.36) **	2.19 (1.22–3.91) **
Marital/cohabiting status				
Single			Reference	Reference
Married/cohabiting			1.81 (0.76–4.34)	1.16 (0.54–2.47)
Parity				
Low (1–2)			Reference	Reference
Average (3–4)			0.92 (0.59–1.42)	1.11 (0.71–1.73)
High (5+)			1.22 (0.79–1.85)	1.25 (0.70–2.23)
Exposure family planning media				
Not exposed			Reference	Reference
Exposed			1.78 (1.25–2.55) **	2.25 (1.51–3.35) ***
Household wealth index				
Poorest			Reference	Reference
Poorer			0.59 (0.31–1.15)	2.05 (1.31–3.22) **
Middle			0.78 (0.41–1.51)	2.23 (1.06–4.72) *
Richer			0.95 (0.49–1.84)	3.56 (1.69–7.48) **
Richest			0.87 (0.36–2.12)	4.39 (1.85–10.47) **
**Community-level variables**				
Place of residence				
Rural			Reference	Reference
Urban			1.48 (0.87–2.52)	1.02 (0.65–1.59)
Region				
North Central			Reference	Reference
North East			0.53 (0.31–0.92) *	0.23 (0.11–0.49) ***
North West			0.23 (0.15–0.37) ***	0.31 (0.16–0.59) **
South East			0.36 (0.19–0.69) *	0.52 (0.22–1.22)
South South			0.36 (0.20–0.66) **	0.63 (0.29–1.38)
South West			0.42 (0.22–0.78) **	0.77 (0.34–1.78)
Community-level literacy^†^			**1.06 (1.02–1.09)** **	**1.14 (1.09–1.19)** ***
Community-level poverty^†^			1.01 (1.00–1.02) *	1.02 (1.01–1.03) **
Community-level exposure to FP media^†^			1.01 (0.98–1.05)	0.99 (0.96–1.03)
**Random effects**	**Null Model**		**Model 4**	
Cluster-level variance (SE)	1.82 (0.32)	3.34 (0.40)	1.28 (0.21)	1.37 (0.34)
ICC (%)	35.6	50.4	27.96	29.36
Explained variance (PCV, %)	-	-	29.76	59.12
**Model summary**				
AIC	19,671.50		18,329.02	
BIC	19,690.68		18,587.87	
Deviance	19,663.50		18,221.02	
	**Model I ^b^**		**Model II ^c^**	
	Contemplation	Action	Contemplation	Action
**Fixed effects intercept** ^‡^	1.04	0.99	0.12	0.04
	**OR [95% CI]**		**aOR [95% CI]**	
PMA survey round/year				
Round 3/2016			Reference	Reference
Round 4/2017			1.47 (0.93–2.33)	1.34 (0.86–2.07)
Round 5/2018			1.23 (0.79–1.91)	1.44 (0.92–2.28)
**Individual-/household-level variables**				
Pregnancy intention				
Intended	Reference	Reference	Reference	Reference
Mistimed	1.48 (1.02–2.13) *	1.94 (1.38–2.71) **	1.59 (1.12–2.27) *	2.17 (1.49–3.17) ***
Unwanted	1.12 (0.49–2.54)	1.55 (0.98–2.46)	1.54 (0.76–3.13)	2.06 (1.36–3.12) **
Maternal age, y				
15–24			Reference	Reference
25–34			2.52 (1.27–4.94) **	3.03 (1.69–5.45) **
35–49			1.49 (0.83–2.68)	2.44 (1.35–4.43) **
Education				
Less than secondary			Reference	Reference
Secondary			2.43 (1.69–3.48) ***	2.09 (1.44–3.02) ***
Higher than secondary			2.69 (1.41–5.16) **	2.94 (1.62–5.35) **
Marital/cohabiting status				
Single			Reference	Reference
Married/cohabiting			1.56 (0.65–3.75)	0.94 (0.44–2.00)
Parity				
Low (1–2)			Reference	Reference
Average (3–4)			0.87 (0.56–1.36)	1.03 (0.66–1.61)
High (5+)			1.03 (0.68–1.55)	0.99 (0.55–1.77)
Exposure family planning media				
Not exposed			Reference	Reference
Exposed			1.73 (1.23–2.45) **	2.08 (1.43–3.01) **
Household wealth index				
Poorest			Reference	Reference
Poorer			0.68 (0.34–1.34)	**2.81 (1.79–4.40)**
Middle			0.99 (0.50–1.94)	**3.55 (1.88–6.69)**
Richer			1.24 (0.61–2.53)	**5.90 (3.15–11.07)**
Richest			1.21 (0.51–2.88)	**8.00 (3.87–16.55)**
**Community-level variables**				
Place of residence				
Rural				
Urban				
Region				
North Central				
North East				
North West				
South East				
South South				
South West				
Community-level literacy^†^				
Community-level poverty^†^				
Community-level exposure to FP media^†^				
**Random effects**	**Model I**		**Model II**	
Cluster-level variance (SE)	1.87 (0.33)	3.49 (0.42)	1.51 (0.25)	1.84 (0.29)
ICC (%)	36.26	51.54	31.4	35.9
Explained variance (PCV, %)	−2.98	−4.63	17.06	44.92
**Model summary**				
AIC	19,617.17		18,542.64	
BIC	19,655.52		18,715.21	
Deviance	19,601.17		18,470.64	
	**Model III ^d^**			
	Contemplation	Action		
**Fixed effects intercept** ^‡^	5.42	11.39		
	**aOR [95% CI]**			
PMA survey round/year				
Round 3/2016				
Round 4/2017				
Round 5/2018				
**Individual-/household-level variables**				
Pregnancy intention				
Intended	Reference	Reference		
Mistimed	**1.57 (1.21–2.20)**	**2.08 (1.50–2.89)**		
Unwanted	1.12 (0.50–2.49)	1.57 (0.99–2.48)		
Maternal age, y				
15–24				
25–34				
35–49				
Education				
Less than secondary				
Secondary				
Higher than secondary				
Marital/cohabiting status				
Single				
Married/cohabiting				
Parity				
Low (1–2)				
Average (3–4)				
High (5+)				
Exposure family planning media				
Not exposed				
Exposed				
Household wealth index				
Poorest				
Poorer				
Middle				
Richer				
Richest				
**Community-level variables**				
Place of residence				
Rural	Reference	Reference		
Urban	1.28 (0.77–2.14)	1.01 (0.64–1.58)		
Region				
North Central	Reference	Reference		
North East	0.67 (0.39–1.13)	0.30 (0.15–0.63) **		
North West	0.29 (0.19–0.46) ***	0.38(0.20–0.71) **		
South East	0.43 (0.23–0.78) **	0.60 (0.27–1.34)		
South South	0.47 (0.26–0.83) **	0.84 (0.40–1.78)		
South West	0.54 (0.31–0.95) *	0.97 (0.44–2.14)		
Community-level literacy^†^	1.06 (1.03–1.09) ***	1.15 (1.11–1.19) ***		
Community-level poverty^†^	1.00 (0.99–1.01)	1.00 (0.99–1.01)		
Community-level exposure to FP media^†^	1.03 (1.00–1.06) *	1.02 (0.99–1.05)		
**Random effects**	**Model III**			
Cluster-level variance (SE)	1.11 (0.19)	1.28 (0.22)		
ICC (%)	25.17	28.04		
Explained variance (PCV, %)	39.11	61.67		
**Model summary**				
AIC	18,926.22			
BIC	19,050.85			
Deviance	18,874.22			

Notes: Reference outcome level = Precontemplation (*n* = 2696); Estimation Method = Pseudo-maximum likelihood; Containment degrees of freedom; Probability distribution = multinomial; Link function = generalized logit. Sample size (individual/household), *N* = 8014; Sample size (community), *N* = 892. Abbreviations: OR–odds ratio, CI–confidence interval, ICC-Intraclass correlation coefficient, PCV– Proportional Change in Variance, AIC–Akaike Information Criteria, FP = Family Planning, Estimation Method = Quadrature, Scaling method 2 used for level 1. ^a^ Best fitting model. ^b^ Model I—includes the main explanatory variable (pregnancy intention). ^c^ Model II—Model I adjusted for only individual/household-level characteristics. ^d^ Model III—Model I adjusted for community-level characteristics. ^e^ Model IV—Model I adjusted for individual/household-level and community-level characteristics (full model). ^†^ Variables were grand-mean centered. ^‡^ Estimates presented as exponents of the log odds (i.e., odds). Values in bold significant at *p* < 0.05; *** *p* < 0.001, ** *p* < 0.01, * *p* < 0.05.

**Table 4:** Odds ratios from multilevel multinomial logistic regression analyses assessing contraceptive method effectiveness level, by individual/household- and community-level factors, according to model.

Characteristics	Null Model ^a^			Model IV ^e^		
	Less Effective	Moderately Effective	Most Effective	Less Effective	Moderately Effective	Most Effective
**Fixed effects intercept**	0.11	0.10	0.04	0.04	0.48	0.01
				**aOR [95% CI]**		
PMA survey round/year						
Round 3/2016				Reference	Reference	Reference
Round 4/2017				1.45 (0.86–2.27)	0.72 (0.47–1.09)	1.31 (0.69–2.51)
Round 5/2018				1.65 (0.97–2.79)	0.69 (0.42–1.13)	1.47 (0.83–2.63)
**Individual-/household-level variables**						
Pregnancy intention						
Intended				Reference	Reference	Reference
Mistimed				1.51 (0.89–2.56)	1.47 (1.02–2.12) *	2.45 (1.41–4.26) **
Unwanted				1.07(0.60–1.92)	0.56 (0.26–1.21)	6.19 (2.04–18.83) **
Maternal age, y						
15–24				Reference	Reference	Reference
25–34				0.96 (0.64–1.44)	1.63 (0.82–3.20)	4.66 (1.70–12.69) **
35–49				1.38 (0.80–2.37)	1.01 (0.51–1.98)	4.89 (1.05–22.76) *
Education						
Less than secondary				Reference	Reference	Reference
Secondary				1.96 (1.27–2.99) **	0.66 (0.39–1.12)	1.18 (0.70–1.98)
Higher than secondary				2.66 (1.51–4.67) **	0.65 (0.25–1.64)	1.69 (0.74–3.84)
Marital/cohabiting status						
Not married				Reference	Reference	Reference
Married				0.71 (0.36–1.39)	0.66 (0.24–1.88)	2.28 (0.73–7.17)
Parity						
Low (1–2)				Reference	Reference	Reference
Average (3–4)				1.06 (0.71–1.58)	1.26 (0.79–2.03)	1.20 (0.32–4.55)
High (5+)				1.05 (0.61–1.78)	1.20 (0.73–1.98)	1.19 (0.24–5.90)
Household wealth index						
Poorest				Reference	Reference	Reference
Poorer				0.83(0.42–1.62)	2.78 (1.69–4.55) ***	3.99 (1.78–8.94) **
Middle				1.14 (0.49–2.64)	2.62 (0.97–7.08)	2.96 (1.32–6.66) *
Richer				1.53 (0.62–3.75)	4.76 (1.69–13.45) **	2.91 (1.30–6.52) *
Richest				2.04 (0.81–5.11)	3.74 (1.99–11.64) *	6.28 (2.42–16.93) **
**Community-level variables**						
Place of residence						
Rural				Reference	Reference	Reference
Urban				1.57 (0.81–3.04)	0.81 (0.45–1.46)	0.59 (0.28–1.25)
Region						
North Central				Reference	Reference	Reference
North East				0.62 (0.24–1.68)	0.38 (0.17–0.84) **	0.13 (0.04–0.41) **
North West				0.34 (0.15–0.76) **	0.91 (0.51–1.62)	0.64 (0.25–1.61)
South East				3.84 (1.57–9.42) **	0.28 (0.12–0.64) **	0.17 (0.05–0.53) **
South South				3.52 (1.50–8.26) **	0.43 (0.20–0.90) **	0.18 (0.06–0.53) **
South West				2.58 (1.06–6.29) *	0.78 (0.0.36–1.71)	**0.22 (0.07–0.69)**
Community-level literacy^†^				1.03 (0.98–1.08)	**1.15 (1.10–1.19)**	**1.12 (1.08–1.19)**
Community-level poverty^†^				1.00 (0.99–1.03)	**1.02 (1.01–1.03)**	1.00 (0.99–1.02)
Community-level exposure to family planning media^†^				0.99 (0.95–1.03)	0.98 (0.94–1.01)	1.01 (0.96–1.05)
**Random effects**	**Null Model**			**Model IV**		
Cluster-level variance (SE)	4.92 (0.65)	2.02 (0.44)	3.05 (0.56)	1.81 (0.35)	1.43 (0.33)	2.03 (0.35)
ICC (%)	59.92	38.04	48.13	35.48	29.34	38.18
Explained variance (PCV, %)	-	-	-	63.68	34.14	42.20
**Model fit summary**						
AIC	17,214.18			15,824.82		
BIC	17,242.94			16,213.09		
Deviance	17,202.18			15,662.82		
**Characteristics**	**Model I ^b^**			**Model II ^c^**		
	**Less effective**	**Moderately effective**	**Most effective**	**Less effective**	**Moderately effective**	**Most effective**
**Fixed effects intercept**	0.10	0.09	0.02	0.01	0.03	0.0004
				**aOR [95% CI]**		
PMA survey round/year						
Round 3/2016				Reference	Reference	Reference
Round 4/2017				1.49 (0.88–2.52)	0.77 (0.49–1.22)	1.43 (0.74–2.79)
Round 5/2018				1.71 (0.95–3.08)	0.75 (0.46–1.23)	1.81 (0.98–3.37)
**Individual-/household-level variables**						
Pregnancy intention						
Intended	Reference	Reference	Reference	Reference	Reference	Reference
Mistimed	1.27 (0.67–2.03)	1.36 (0.90–2.05)	2.27 (1.36–3.78) **	1.46 (0.85–2.51)	1.47 (1.03–2.11) *	2.45 (1.39–4.29) **
Unwanted	1.04 (0.58–1.86)	0.50 (0.24–1.05)	7.29 (1.64–32.44) **	1.24(0.69–2.23)	0.60 (0.27–1.34)	6.90 (2.28–20.93) **
Maternal age, y						
15–24				Reference	Reference	Reference
25–34				1.16 (0.77–1.77)	1.72 (0.89–3.33)	4.66 (1.73–12.56) **
35–49				1.81 (1.04–3.15)	1.12 (0.59–2.14)	4.98 (1.10–22.51) *
Education						
Less than secondary				Reference	Reference	Reference
Secondary				2.53 (1.69–3.77) ***	0.84 (0.48–1.46)	1.56 (0.91–2.66)
Higher than secondary				3.37 (1.97–5.76) ***	0.85 (0.32–2.24)	2.31 (0.99–5.37)
Marital/cohabiting status						
Not married				Reference	Reference	Reference
Married				0.60 (0.30–1.19)	0.66 (0.24–1.87)	2.81 (0.74–10.63)
Parity						
Low (1–2)				Reference	Reference	Reference
Average (3–4)				1.03 (0.68–1.54)	1.22 (0.76–1.97)	1.15 (0.30–4.34)
High (5+)				0.81 (0.46–1.42)	1.11 (0.67–1.84)	1.18 (0.25–5.69)
Exposure family planning media						
Not exposed				Reference	Reference	Reference
Exposed				1.46 (0.99–213)	2.14 (1.36–3.36) **	1.12 (0.64–1.95)
Household wealth index						
Poorest				Reference	Reference	Reference
Poorer				1.92 (1.02–3.58) *	2.91 (1.77–4.76) ***	4.46 (2.07–9.63) **
Middle				3.97 (2.04–7.72) **	2.53 (1.11–5.74) *	3.27 (1.47–7.29) **
Richer				5.88 (3.14–11.02) ***	4.32 (1.83–10.19) **	2.95 (1.26–6.90) *
Richest				8.63 (4.42–16.86) ***	3.33 (1.34–8.38) **	6.53 (2.75–15.51) ***
**Community-level variables**						
Place of residence						
Rural						
Urban						
Region						
North Central						
North East						
North West						
South East						
South South						
South West						
Community-level literacy^†^						
Community-level poverty^†^						
Community-level exposure to family planning media^†^						
**Random effects**	**Model I**			**Model II**		
Cluster-level variance (SE)	4.98 (0.66)	2.10 (0.45)	3.52 (0.55)	2.56 (0.49)	1.86 (0.42)	2.64 (0.49)
ICC (%)	60.23	38.98	51.66	43.72	36.11	44.53
Explained variance (PCV, %)	−1.31	−4.05	−15.16	48.02	7.96	13.46
**Model fit summary**						
AIC	17,031.50			16,162.42		
BIC	17,089.02			16,421.27		
Deviance	17,007.50			16,054.42		
**Characteristics**	**Model III ^d^**					
	**Less effective**	**Moderately effective**	**Most effective**			
**Fixed effects intercept^‡^**	0.13	0.80	0.45			
	**aOR [95% CI]**					
PMA survey round/year						
Round 3/2016						
Round 4/2017						
Round 5/2018						
**Individual-/household-level variables**						
Pregnancy intention						
Intended	Reference	Reference	Reference			
Mistimed	1.43 (0.89–2.31)	1.44 (0.95–2.17)	2.35 (1.41–3.89) **			
Unwanted	1.03 (0.58–1.84)	0.50 (0.25–1.03)	6.39 (1.44–28.41) *			
Maternal age, y						
15–24						
25–34						
35–49						
Education						
Less than secondary						
Secondary						
Higher than secondary						
Marital/cohabiting status						
Not married						
Married						
Parity						
Low (1–2)						
Average (3–4)						
High (5+)						
Exposure family planning media						
Not exposed						
Exposed						
Household wealth index						
Middle						
Richer						
Richest						
**Community-level variables**						
Place of residence						
Rural	Reference	Reference	Reference			
Urban	1.46 (0.77–2.75)	0.97 (0.52–1.79)	0.67 (0.26–1.68)			
Region						
North Central	Reference	Reference	Reference			
North East	0.67 (0.26–1.68)	0.38 (0.18–0.83) *	0.18 (0.05–0.59) **			
North West	0.35 (0.17–0.75) **	0.95 (0.53–1.69)	0.78 (0.27–2.20)			
South East	3.32 (1.45–7.58) **	0.34 (0.15–0.77) **	0.19 (0.05–0.66) *			
South South	3.55 (1.63–7.75) **	0.47 (0.22–1.01) *	0.21 (0.06–0.66) *			
South West	2.57 (1.12–5.88) *	0.78 (0.36–1.71)	0.27 (0.08–0.89) *			
Community-level literacy^†^	1.05 (1.00–1.09)	1.12 (1.08–1.16) ***	1.14 (1.09–1.19) ***			
Community-level poverty^†^	0.99 (0.98–1.00) *	1.01 (0.99–1.02)	0.99 (0.98–1.01)			
Community-level exposure to family planning media^†^	1.01 (0.97–1.05)	0.99 (0.97–1.03)	1.01 (0.97–1.04)			
**Random effects**	**Model III**					
Cluster-level variance (SE)	1.70 (0.31)	1.38 (0.32)	2.11 (0.35)			
ICC (%)	34.01	29.61	39.09			
Explained variance (PCV, %)	65.96	34.14	39.92			
**Model fit summary**						
AIC	16,319.81					
BIC	16,506.75					
Deviance	16,241.81					

Notes: Reference outcome level = No method (*n* = 5829); Estimation Method = Pseudo-maximum likelihood; Containment degrees of freedom; Probability distribution = multinomial; Link function = generalized logit; Estimation Method = Quadrature; Scaling method 2 used for level 1. Abbreviations: OR–odds ratio, CI–confidence interval, ICC-Intraclass correlation coefficient, PCV– Proportional Change in Variance, AIC–Akaike Information Criteria, FP = Family Planning. Values in bold significant at *p* < 0.05; *** *p* < 0.001, ** *p* < 0.01, * *p* < 0.05. ^a^ Best fitting model. ^b^ Model I—includes the main explanatory variable (pregnancy intention). ^c^ Model II—Model I adjusted for only individual/household-level characteristics. ^d^ Model III—Model I adjusted for community-level characteristics. ^e^ Model IV—Model I adjusted for individual/household-level and community-level characteristics (full model). ^†^ Variables were grand-mean centered. ^‡^ Estimates presented as exponents of the log odds (i.e., odds).

## Data Availability

The data used for this study is publicly available and can be downloaded from https://www.pmadata.org (accessed on 15 January 2023).
